# SiteSeek: Post-translational modification analysis using adaptive locality-effective kernel methods and new profiles

**DOI:** 10.1186/1471-2105-9-272

**Published:** 2008-06-10

**Authors:** Paul D Yoo, Yung Shwen Ho, Bing Bing Zhou, Albert Y Zomaya

**Affiliations:** 1 Advanced Networks Research Group, School of Information Technologies (J12), The University of Sydney, Sydney, NSW 2006, Australia; 2 Retroviral Genetics Laboratory, Centre for Virus Research, Westmead Millennium Institute, Westmead Hospital, The University of Sydney, Westmead, NSW 2145, Australia; 3 Sydney Bioinformatics Centre and the Centre for Mathematical Biology,The University of Sydney, Sydney, NSW 2006, Australia

## Abstract

**Background:**

Post-translational modifications have a substantial influence on the structure and functions of protein. Post-translational phosphorylation is one of the most common modification that occur in intracellular proteins. Accurate prediction of protein phosphorylation sites is of great importance for the understanding of diverse cellular signalling processes in both the human body and in animals. In this study, we propose a new machine learning based protein phosphorylation site predictor, SiteSeek. SiteSeek is trained using a novel compact evolutionary and hydrophobicity profile to detect possible protein phosphorylation sites for a target sequence. The newly proposed method proves to be more accurate and exhibits a much stable predictive performance than currently existing phosphorylation site predictors.

**Results:**

The performance of the proposed model was compared to nine existing different machine learning models and four widely known phosphorylation site predictors with the newly proposed PS-Benchmark_1 dataset to contrast their accuracy, sensitivity, specificity and correlation coefficient. SiteSeek showed better predictive performance with 86.6% accuracy, 83.8% sensitivity, 92.5% specificity and 0.77 correlation-coefficient on the four main kinase families (CDK, CK2, PKA, and PKC).

**Conclusion:**

Our newly proposed methods used in SiteSeek were shown to be useful for the identification of protein phosphorylation sites as it performed much better than widely known predictors on the newly built PS-Benchmark_1 dataset.

## Background

Post-translational modifications are observed on almost all proteins analysed to date. During phosphorylation, a phosphate molecule is placed on another molecule resulting in the functional activation or inactivation of the receiving molecule. These modifications have a substantial influence on the structure and functions of protein. Phosphorylation at the serine, threonine and tyrosine residues by enzymes of the kinase and phosphatise super-families is one of the most frequent forms of post-translational modifications in intracellular proteins. As phosphorylation has a significant impact on diverse cellular signalling processes, it is needed in the regulation of cell differentiation, as a trigger for the progression of the cell cycle and control of metabolism, transcription, apoptosis, cytoskeletal rearrangements [1-7] in animals. As importantly, the phosphorylation of protein is considered as being a key event in many signal transduction pathways of biological systems [8]. It is thus important for us to be able to accurately determine the phosphorylation state of proteins so as to better identify the *state *of a cell.

It has been widely reported in literature that a large number of human diseases are caused by a disruption of normal cellular phosphorylation events. For example, phosphorylated tyrosines are recognised by specialised binding domains on other proteins, and such interactions are used to initiate intracellular signalling pathways. Aberrant tyrosine phosphorylation is a hallmark of many types of cancer. Tyrosine phosphorylation also plays major roles in cellular physiology, and functional perturbation of protein-tyrosine kinases and protein-tyrosine phosphatases underlie many human diseases [9].

In order to determine phosphoproteins and individual phosphorylation sites, various experimental tools have been used. However, *in vivo *or *in vitro *identification of phosphorylation sites is labour-intensive, time-consuming and often limited to the availability and optimisation of enzymatic reactions [7,8,10]. Several large-scale phosphoproteomic data using the mass-spectrometry approach have been collected and published [11-13] but are still not helpful in distinguishing the kinase-specific sites on substrates. For example, mass spectrometry methods have been shown to be disfavourable in the identification of phosphate-modified residues, leading to an underestimation of the extent of phosphorylation presents *in vivo *[14].

Due to the practical limitations and complexities of the previously-mentioned methods, many scientists have turned to computer-based methods. Computer-based methods can efficiently handle massive amounts of protein data, determine phosphoprotiens and identify individual phosphorylation sites from one dimensional atomic coordinates with high precision. Several computer-simulated machine learning techniques such as Artificial Neural Networks (ANNs) and Support Vector Machines (SVMs) have been extensively used in various biological sequence analyses as well as phosphorylation site prediction. These methods are built based on the assumption that neighbouring residues to the phosphorylated site represents the main determinant for kinase specificity [10,15].

Although a large number of machine learning based methods have proved to be effective in the prediction of phosphorylation sites, several important issues that could potentially degrade the performance of machine learning or statistical-based methods have been largely ignored. The high dimensionality of protein sequence data not only causes a dynamic increase in computational complexity but also creates an overfitting/generalisation problem for non-parametric methods. With machine learning models, better generalisation and faster training (computationally efficient) can be achieved when they have fewer weights to be adjusted with fewer inputs.

This study aims to develop an accurate and stable phosphorylation site predictor. Our proposed model named, SiteSeek uses a semi-parametric form of a state-of-the-art machine learning model dubbed, Adaptive Locality-Effective Kernel Machine (Adaptive-LEKM). In addition, with the boosting algorithm, it adaptively combines the learners to find an optimised fit for the given phosphoprotiens. To efficiently capture suitable information from protein sequences, it uses a newly developed Compact Evolutionary and Hydrophobic Profile (CEH-Profile) built on Position Specific Scoring Matrix (PSSM) and Simultaneously Axially and Radially Alignment Hydrophobicity (SARAH1) Hydrophobicity scale. In our experiments, the SiteSeek excels in efficiently processing high dimensional protein data with a more accurate and stable predictive performance than currently existing models. The novel feature of this study is the use of a new machine learning based semi-parametric model, a newly developed profile (CEH-Profile) and the use of unique training dataset (PS-Benchmark_1) that contains experimentally verified phosphorylation sites manually extracted from major protein sequence databases and the literature.

## Results

Our experiments consist of four consecutive steps. First, we demonstrate the usefulness of our proposed CEH-Profile by comparing its prediction accuracy with four other well-known amino acids encoding methods. Second, the predictive performance of our proposed machine learning model, Adaptive-LEKM, specially designed for the high dimensional problem of protein sequence data is compared with other nine contemporary machine learning models for prediction accuracy, sensitivity, specificity, correlation-coefficient, type I and type II errors on newly built PS-Benchmark_1 dataset. Next, we analyse the result from SiteSeek which uses the novel Adaptive-LEKM and the CEH-Profile when tested on four main kinase groups and four main kinase families. We then compare those results with the consensus results from literature. Lastly, the predictive performance of SiteSeek is directly compared with thee of the most widely known contemporary phosphorylation site predictors on PS-Benchmark_1 dataset.

### Compact evolutionary and hydrophobicity profile

In order to prove the usefulness of CEH-Profile, the suitability of the newly proposed profile to the prediction of phosphorylation sites is compared with other four most widely used encoding schemes (PSSM, SARAH1, Hydrophobicity Scale and Orthogonal Encoding). As shown in Table 1, the CEH-Profile and CompactPSSM showed fairly improved performance for the prediction of phosphorylation sites. With CompactPSSM, we obtain 3% increased prediction accuracy than the widely used PSSM. Furthermore, CEH-Profile which additionally uses hydrophobicity in the format of effective 5 bit binary representation (SARAH1 Scale) has a better accuracy at 5.1% – 6.5% than original PSSM. The sensitivity of CEH-Profile has significantly increased by 5% from the original PSSM. It seems that with CEH-Profile, we can combine the advantage of using both PSSM and SARAH1 profiles as SARAH1 provides a good model sensitivity level of 0.80.

**Table 1:** Comparison of encoding schemes.

Models	Accuracy (Ac)	Sensitivity (Sn)	Specificity (Sp)	Correlation-Coefficient (Cc)	Type I ER	Type II ER
**CEH-Profile**	**0.75**	**0.74**	**0.76**	**0.52**	**0.11**	**0.13**
CompactPSSM	0.73	0.71	0.75	0.46	0.13	0.14
PSSM	0.70	0.69	0.71	0.40	0.15	0.15
OE	0.58	0.60	0.56	0.16	0.23	0.19
Hydrophobicity	0.58	0.56	0.61	0.17	0.19	0.23
SARAH1	0.61	0.80	0.42	0.24	0.29	0.10

This experiment based on our hypothesis proved that less discriminatory features that reside in original PSSM can be replaced by an effective hydrophobicity scale so that a new profile which contains compact evolutionary information as well as hydrophobic values can be created. As examined in *Discussion*, several limitations of existing PSSM and OE profiles can be effectively minimised by utilising the novel CEH-Profile. Evidently, CEH-Profile is more useful than widely known sequence profiles for protein phosphorylation site prediction.

### Comparison of Adaptive-LEKM with other machine learning models

The predictive performance of Adaptive-LEKM was compared with nine other existing state-of-the-art machine learning models such as General Regression Neural Network (GRNN), Radial Basis Neural Network (RBFN), Multi-Layered Perceptron (MLP), kernel Nearest Neighbour (kNN), Decision Tree (J48), kernel Logistic Regression (KLR), and three different transductive SVMs, namely SVM^*light*^, AdaBoost-SVM and Locality-Effective Kernel Machine (LEKM). Table 2 shows the evaluation results of each model in terms of Accuracy (Ac), Sensitivity (Sn), Specificity (Sp), Correlation-Coefficient (Cc), Variance (Var) and Time on PS-Benchmark_1 dataset.

**Table 2:** Prediction results of machine learning models on PS-Benchmark_1 dataset.

Models	Accuracy (Ac)	Sensitivity (Sn)	Specificity (Sp)	Correlation-Coefficient (Cc)	Var.	Time
**Ada-LEKM**	**0.823**	**0.801**	**0.845**	**0.646**	**0.022**	**35.745**
Ada-SVM	0.791	0.776	0.806	0.583	0.031	51.593
SVM	0.798	0.787	0.809	0.596	0.030	32.886
**LEKM**	**0.783**	**0.773**	**0.792**	**0.565**	**0.020**	**22.421**
kNN	0.767	0.753	0.781	0.534	0.032	35.630
GRNN	0.759	0.724	0.793	0.518	0.041	85.422
MLP	0.752	0.715	0.789	0.505	0.046	180.344
RBFN	0.737	0.685	0.788	0.475	0.044	68.654
DT (J48)	0.732	0.718	0.747	0.465	0.025	8.393
KLR	0.726	0.682	0.772	0.456	0.038	156.690

As shown in Table 2, one of our models, LEKM successfully reached the best model stabilisation (Var: 0.020) with less computational requirements (Time: 22.421). However, one of the methods used in LEKM (semi-parametric approximation) showed a slightly less accurate learning. Hence, we utilised AdaBoost algorithm for the fine tuning of the LEKM and it (Adaptive-LEKM) finally achieved the best accuracy with a fair level of model stableness and furthermore reduced complexity. In addition, Adaptive-LEKM achieved much better model robustness than other methods with the Cc of 0.39. Our methods used in Adaptive-LEKM, semi-parametric approximation and adaptive tuning of the model using AdaBoost were confirmed to be more suitable in processing high dimensional protein data than other non-parametric models. It should be noted that the AdaBoost algorithm was also tested with the original SVM, but no significant improvement was observed (Table 2).

**Figure 1:**
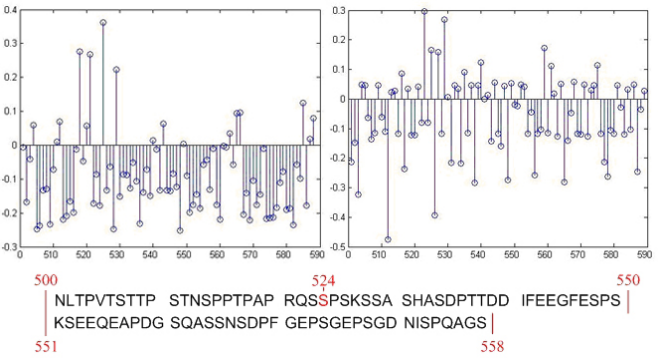
Prediction scores simulated by Adaptive-LEKM and SVM.

Figure 1 shows the comparison of prediction scores simulated by the Adaptive-LEKM and the original SVM on a protein chain (Swiss-Prot Entry: O75553). The protein chain has 588 residues with two tyrosine and one serine sites at the residue 198, 220 and 524 respectively. As shown in Figure 1, SVM's signal at the site is generally 0.3 point with many fluctuating neighbouring signals making the site undistinguished. On the other hand, Adaptive-LEKM provides very clearer indications of the phosphorylation site at the residue 524 and its signal is generally stronger than that of other methods (0.37921 point). Thus, Adaptive-LEKM offers an additional advantage over other machine learners with a clearer and stronger indication of site locations.

### Predictive performance of SiteSeek on major kinase families and groups

Here we look at experimental results obtained by SiteSeek on four main kinase groups and four main kinase families in terms of Ac, Sn, Sp, Cc, Type I and Type II ERs. SiteSeek uses the Adaptive-LEKM and is trained with the novel CEH-Profile. Table 3 compares the results of SiteSeek with the consensus results of the literature. In general, SiteSeek showed a 9% increase in prediction accuracy than the consensus results. As for model stableness, SiteSeek also achieved a fairly low level of average variance in four evaluation measures (0.041, 0.074, 0.019, 0.083). The sensitivity of SiteSeek on CDK, PKA and PKC kinase families are distinguishably higher than the consensus results. This means that stable prediction capability of our model comes with effectively reducing the false negative values (Type I ER). Type I ER indicates experimentally verified unmodified sites that are predicted (incorrectly) to be modified.

**Table 3:** Prediction results of Adaptive-LEKM for the four kinase families.

K-Families	Accuracy (Ac)	Sensitivity (Sn)	Specificity (Sp)	Correlation-Coefficient (Cc)	Type I ER	Type II ER
CDK	**0.909**	**0.895**	**0.921**	**0.817**	**0.043**	**0.046**
	0.777	0.455	0.992	0.900		
CK2	**0.918**	**0.881**	**0.948**	**0.835**	**0.029**	**0.051**
	0.840	0.765	0.888	0.660		
PKA	**0.891**	**0.843**	**0.929**	**0.779**	**0.039**	**0.069**
	0.816	0.561	0.987	0.640		
PKC	**0.827**	**0.731**	**0.903**	**0.650**	**0.053**	**0.118**
	0.726	0.475	0.898	0.420		

Avg.	**0.886**	**0.838**	**0.925**	**0.770**	**0.041**	**0.071**
	0.790	0.564	0.941	0.655		

Var.	**0.041**	**0.074**	**0.019**	**0.083**	**0.010**	**0.032**
	0.050	0.142	0.056	0.196		

The experimental results of SiteSeek are written in bold and others are the consensus results of literature obtained by Kim et al 2004.

As for the four main kinase groups (Table 4), the overall results are somewhat lower than those of kinase families. The best accuracy was obtained with the CMGC kinase group (Ac: 0.90) whereas the TK kinase group showed the lowest accuracy of 0.79. Compared to the experiments on kinase families, Cc values dropped significantly for 0.9 points. This result may reveal that computational models are more suitable to find stronger correlations among same kinase families than those of kinase groups.

**Table 4:** Prediction results of Adaptive-LEKM for the four kinase groups.

K-Gruops	Accuracy (Ac)	Sensitivity (Sn)	Specificity (Sp)	Correlation-Coefficient (Cc)	Type I ER	Type II ER
AGC	0.862	0.796	0.913	0.719	0.048	0.090
CAMK	0.821	0.721	0.900	0.638	0.056	0.123
CMGC	0.900	0.891	0.907	0.796	0.054	0.046
TK	0.792	0.667	0.892	0.580	0.060	0.148

Avg.	0.844	0.769	0.903	0.683	0.055	0.102
Var.	0.047	0.097	0.009	0.094	0.005	0.044

### Comparison with other contemporary phosphorylation site predictors

The predictive performance of our proposed predictor, SiteSeek which uses above-mentioned Adaptive-LEKM and CEH-Profile is compared with four contemporary phosphorylation site predictors on same testing dataset, PS-Benchmark_1. DISPHOS [16] is trained on over 2000 non-redundant experimentally confirmed protein phosphorylation sites and uses position-specific amino acid frequencies as well as disorder information to improve the discrimination between phosphorylation and non-phosphorylation sites. The prediction accuracy of DISPHOS reaches 81.3% for Serine, 74.8% for Threonine, and 79.0% for Tyrosine. However, DISPHOS provides little information about the corresponding protein kinases for the predicted phosphorylation sites. Scansite [17] identifies short protein motifs that are recognised by phosphorylation protein serine/threonine or tyrosine kinases. Each motif used in Scansite was constructed from a set of experimentally validated phosphorylation sites and was represented as a position-specific scoring matrix. NetPhosK [18] is an enhanced version of NetPhos uses an artificial neural network and incorporates the functionality of providing protein kinases (PKs) information for 17 PKs. In the identification of protein kinase A (PKA) phosphorylation sites, NetPhosK achieves 100% sensitivity and 40% specificity. Finally, PredPhospho [19] uses support vector machine and demonstrates a superior predictive performance of 83–95% at the kinase family level, and 76–91% at the kinase group level.

Table 5 shows the comparison of our proposed model with the above-mentioned four contemporary phosphorylation site predictors on the PS-Benchmark_1 dataset. SiteSeek has shown the best overall accuracy amongst the state-of-the-art predictors by reaching a prediction accuracy of 86.6%. Interestingly, the sensitivity of SiteSeek was far more superior to other predictors with 84.4%. In addition, its model robustness was again confirmed as Cc value showed 8.3 points higher than widely known Scansite. This result corresponds with the previous experimental results in that the newly proposed CEH-Profile provides more useful information to the predictors for kinase family level prediction. In addition, the results give evidence that the semi-parametric approach of Adaptive-LEKM which brings a more stable prediction is a better for phosphorylation site prediction than current existing methods. The prediction ability of the proposed model, which sustains its stability, was again proved with PS-Benchmark_1 dataset.

**Table 5:** Predictive performance of phosphorylation site predictors.

	Accuracy (Ac)	Sensitivity (Sn)	Specificity (Sp)	Correlation-Coefficient (Cc)	Type I Error	Type II Error
**SiteSeek**	**0.866**	**0.844**	**0.885**	**0.730**	**0.063**	**0.071**
PredPhospho	0.843	0.821	0.862	0.684	0.076	0.079
NetPhosK	0.836	0.790	0.876	0.670	0.066	0.099
Scansite	0.827	0.755	0.883	0.647	0.066	0.107
DISPHOS	0.805	0.773	0.827	0.601	0.092	0.106

Although each predictor was trained using its own training dataset, they all were tested on same benchmark dataset, (PS-Benchmark_1) which contains 1,668 polypeptide chains (Refer to Section "PS-Benchmark_1).

## Discussion

Over the past decades, many computational prediction algorithms have been developed for various proteomic studies. They have evolved from simple linear statistics to complex machine learners. However, the most significant breakthroughs were the incorporation of new biological information into an efficient prediction model and the development of new models which can efficiently exploit suitable information from its primary sequence. For example, the exploitation of evolutionary information that is available from protein families has brought significant improvements in the prediction of protein secondary structure (about 6–8%) [20-24].

Compared to protein structure predictions, a small number of studies to find suitable information/representations for phosphorylation site prediction have been reported. Like protein secondary structure prediction, most of the phosphorylation site prediction methods also use the evolutionary information in the format of PSSM (sequence profile) [16-18,25]. The theory behind using the sequence profile is based on the fact that the sequence alignment of homologous proteins accords with their structural alignment and aligned residues usually have similar structures. Thus, the sequence profile can provide more information about structure than the single sequence to its learner.

Although the sequence profile provides more structural information, the structural information that resides in the sequence profile may not be a significant importance in the case of phosphorylation site prediction. It has been observed that approximately only ten neighbouring residues are major determinants of phosphorylation sites. Many models have been built on this observation and performed reasonably well with a number of specific kinases. However, the specificity determinants and rules remain elusive for a large number of protein kinases that display a number of substrates sharing little or no sequence similarity in the known phosphopeptides [10]. Furthermore, most databases searched by current alignment tools like PSI-BLAST not only contain a number of non-phosphoprotiens, but also generate a large numbers of irrelevant hits from the protein databases [8].

Another commonly used amino acid encoding method is dubbed orthogonal encoding, also known as the binary representation or the distributed encoding. In orthogonal encoding, each letter can be represented by a twenty dimensional binary vector indicating the presence of a particular amino acid type [26]. The twenty standard amino acids are ordered one through twenty, and the *i*^*th *^amino acid has the binary codeword of twenty bits with the *i*^*th *^bit set to "1" and all others to "0"s, for i = 1, 2,..., 20. For example, 'Alanine' is expressed by 0000000000 00000000001, 'Cysteine' is encoded as 0000000000 0000000010 and so on. Among the first twenty units of the vector, each unit stands for one type of amino amid. In order to allow a window to extend over the *N *terminus and the *C *terminus, the 21st unit has to be added. A residue with window size *n *is encoded in 21*(2n+l) bits with the binary code-words of amino acids concatenated based on their order in the window [27]. It has been one of the most widely adopted methods as it does not introduce any artificial correlations between the amino acids.

However, orthogonal encoding has several widely known drawbacks. Firstly, the dimension of residue vector can increase rapidly as *n *increases, it may lead to large computational cost and model complexity (a typical input window of 13 residues requires 567 = (21*(2*13+1) input nodes and connecting weights), and recognition bias. Thus, it can cause poor performance of the classifiers [28-30]. Secondly, the use of Euclidean space has no theoretic foundation in biology or chemistry and hence might reduce the accuracy of a model. According to the numerical assignment in the distributed method, the distance between any two different amino acids is $\sqrt{2}$ and would conflict with reality in biology [29].

The encoding methods discussed above are employed by most well-known protein structure predictors and are shown to be useful as they sufficiently contain information required for general protein structure prediction tasks. However, as phosphorylation site prediction does not only involve various chemical interactions but also is known as a non-structural prediction task, the above-mentioned encoding methods may not be suitable for this problem. In this study, we observed that widely known sequence profile contains irrelevant features which impede accurate recognition by the learner. We thus developed a new profile that replaces the prediction irrelevant features by hydrophobic information represented in SARAH1 scale. This method when used in CEH-Profile showed reasonable improvement over existing profiles.

In the literature, the number of encoding schemes proposed for phosphorylation site prediction is far less than the ones for other proteomic applications. As discussed above, existing methods have shown several critical drawbacks for phosphorylation site prediction. Hence, we emphasise that researchers should devote their effort to seeking a suitable representation of amino acids for phosphorylation site prediction to reach the upper boundary of prediction accuracy.

Several considerable issues have been inevitably raised in this study. Most importantly, the training set used in this study may contain a certain amount of incorrect information as some of non-site residues can be determined as site-residues in the future. The prediction accuracy for general machine learning (non-parametric) models is highly dependent on the quality of the input/training dataset. If the dataset were to contain inaccurate information, it can significantly affect the learning process thus predicting sites incorrectly. The use of a semi-parametric model in this study takes upon assumptions that are stronger than those of non-parametric models so that it can more robustly perform its prediction task on noise-datasets than pure non-parametric methods. However, even when the semi-parametric approach was shown to be more resistant to the noise-data, the problems caused by using potentially corrupted dataset cannot be resolved completely unless an experimentally complete and faultless phosphorylation data is provided.

Despite of our effort to collect high quality phosphorylation data for all kind of kinase families, each kinase dataset is excessively biased to a few kinase families. For example, PKA and PKC kinase family data take approximately 69% of AGC kinase group dataset. Hence, a learner trained with AGC kinase group dataset may not perform well with other kinase families in the AGC kinase group as it was trained mostly with PKA or PKC kinase family data. It is ideal if we can collect more experimentally verified data for those kinase families; however, in the case that this is not possible, *matched sampling *methods were shown to be somewhat effective.

One of the experiments conducted to compare the performance of SiteSeek with other existing phosphorylation site predictors (Table 5) reveals several considerable issues. First, the predictive performance of other predictors can be overestimated in that it is almost infeasible to confirm whether their training datasets contains any of amino acids sequences in our testing set. The presence of testing examples in their training dataset not only violates the assumptions needed for learning but also makes evaluation measures unreliable. Hence, this issue should be taken into account carefully for more accurate evaluation and comparison of models. It should be noted that as the seven-fold cross validation was also used for the evaluation of SiteSeek, no redundant data entry for training and testing sets was found. Second, the window sizes adopted in this study may not be suitable for kinase family or group based predictions. In the literature, it has been discussed that the major determinants for phosphorylation sites are 9 neighbouring residues for tyrosine and threonine; 11 for serine [15]. Several studies using computational methods recently have shown that the optimal window sizes are found to be different depending on types of kinase families. However, no wet-lab research has validated the kinase family based optimal window sizes to date. Hence, further research should be carried out so as to provide for a more accurate evaluation and prediction of phosphorylation sites.

## Conclusion

This paper identified the effectiveness and utility of our newly proposed machine learning based predictor, SiteSeek for phosphorylation site prediction. This study addressed two important issues in the computational prediction of protein phosphorylation sites. Current encoding schemes like PSSM and Orthogonal Encodings do not provide sufficient information for accurate prediction of phosphorylation sites using existing computational models. Our approach uses compact PSSM with efficient hydrophobicity scale proves to be more effective in the prediction of phosphorylation sites. Next, for a given set of high dimensional protein data, the combination of a parametric local model with a non-parametric global model provided a way of fine-tuning the model by the adjustment of a single smoothing parameter *σ *as well as providing efficient semi-parametric approximation. This was demonstrated by our above four consecutive experiments. The semi-parametric approach used in Adaptive-LEKM was shown to be effective by finding an optimal trade-off between parametric and non-parametric models with significantly reduced computations. When tested with the newly built PS-Benchmark_1 dataset, SiteSeek which uses the Adaptive-LEKM and CEH-Profile achieved the best prediction accuracy when compared with contemporary phosphorylation site predictors. Thus, allowing us to accurately predict phosphorylation sites in proteins so as to better understand their functions in biological systems.

## Methods

### PS-Benchmark_1 dataset

The fair comparison and assessment of phosphorylation site predictors is complicated as all use different phosphorylation site datasets in the literature. In this study, we use a newly developed comprehensive dataset, namely PS-Benchmark_1 for the purpose of benchmarking sequence-based phosphorylation site prediction methods. It is widely known that accurate classification is highly dependent upon the quality of data sets of both positive and negative examples. However, such a golden standard datasets are not yet available for protein phosphorylation site prediction. PS-Benchmark_1 contains experimentally verified phosphorylation sites manually extracted from major protein sequence databases and the literature. The dataset comprises of 1,668 polypeptide chains and as shown in Table 6, the chains are categorised in four major kinase groups, namely cAMP-dependent protein kinase/protein kinase G/protein kinase C extended family (AGC), calcium/calmodulin-dependent kinase (CAMK), cyclin-dependent kinase-like kinase (CMGC) and tyrosine kinase (TK) groups. The dataset comprises of 513 AGC chains, 151 CAMK chains, 330 CMGC chains, and 216 TK chains. The dataset is non-redundant in a structural sense: each combination of topologies occurs only once per dataset. Protein sequences are taken from the Protein Data Bank (PDB) [31], Swiss-Prot [32], Phospho3D [10], Phospho.ELM [33] and literature.

**Table 6:** Four main kinase groups.

**AGC Group**	**CAMK Group**	**TK Group**	**CMGC Group**	**Other Group**
DMPK_group	CaM-KIalpha	Abl	CDK_group	CK2 alpha
GRK_group	CaM-KI_group	ALK	CDK1	CK2 beta
GRK-1	CaM-KII_group	Axl	CDK11	CK2_group
GRK-2	CaM-KIIalpha	Csk	CDK2	N/A
GRK-3	CaM-KIV	EGFR	CDK4	
GRK-4	CaM-Kkalpha	EphA2	CDK5	
GRK-5	CaM-Kkbeta	EphA3	CDK6	
GRK-6	CDPK	EphA4	CDK7	
NDR1	CHK1	EphA8	CDK9	
NDR2	CHK2	EphB1	CLK1	
PDK1	DAPK_group	EphB2	DYRK1A	
PDK2	DAPK1	EphB3	DYRK1B	
PDK_alpha	DAPK2	EphB5	DYRK2	
PKA_group	DAPK3	FAK	DYRK3	
PKA alpha	MAPKAPK2	Fer	GSK-3_group	
PKB_group	MARK_group	FGFR_group	GSK-3alpha	
PKB beta	MLCK_group	FGFR1	GSK-3beta	
PKC_group	PHK_group	FGFR3	MAPK_group	
PKC alpha	Pim-1	FGFR4	MAPK1	
PKC beta	PKD1	JAK_group	MAPK10	
PKC delta	PKD2	JAK1	MAPK11	
PKC epsilon	PKD3	JAK2	MAPK12	
PKC eta	RSK_group	JAK3	MAPK13	
PKC gamma	RSK-1	Met	MAPK14	
PKC iota	RSK-2	PDGFR_group	MAPK3	
PKC theta	RSK-3	PDGFR alpha	MAPK4	
PKC zeta	RSK-5	PDGFR beta	MAPK6	
PKG	N/A	Ret	MAPK7	
PKG1		Src	MAPK8	
PKG1A		Src_group	MAPK9	
PKG1B		Syk	PRP4	
PKG2		Tec	N/A	
RSK_group		Tie2		
RSK-1		TRKA		
RSK-2		TRKB		
RSK-3		N/A		
RSK-5				
SGK_group				

### Compact evolutionary and hydrophobicity profile

**Figure 2:**
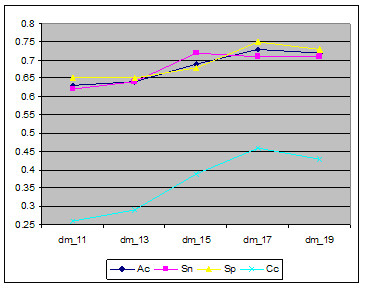
Comparison of different data dimensions.

One of our preliminary experiments showed that the less discriminative features residing in the original sequence profile can diminish the predictive performance of the learners. So, we utilise the non-linear auto-associative network to filter the less-discriminative features in the dataset. In the process of the filtering, the model finds the optimal data dimension for more accurate prediction. As shown in Figure 2, the reduced data dimension of 17 (dm_17) provides better evaluation measures than its original data dimensions of the sequence profile (dm_20). The best data dimension (dm_17) observed in this experiments divulge that not all the information in the sequence profile is useful for accurate prediction. In other words, some amounts of less-discriminative features that can impede recognition of the learner exist in the profile. Hence, it is imperative that to reach the upper boundary of the prediction accuracy, additional informative features from other sources should be added.

The promising additional information can be sought from the widely known hydrophobicity scales. A number of researchers selected hydrophobicity as the main feature among many other physicochemical properties for protein structure prediction (such as polarity, charge or size) [34-36]. More importantly, several recent studies reported that protein hydrophobicity can be affected by the level of phosphorylation or vice versa [37-39]. Hydrophobicity is a very important factor in protein stability. The "hydrophobic effect" is believed to play a fundamental role in the spontaneous folding of proteins. It can be expressed as the free energy (kilocalories per mole) of transfer of amino acid side chains from cyclohexane to water. The amino acids with positive values of free energy in transferring cyclohexane to water are hydrophobic and the ones with negative values are hydrophilic [34]. Table 7 shows hydrophobicity scales, and the hydrophobicity matrix can be formulated using the following function.

**Table 7:** Hydrophobicity Scale: Nonpolar → Polar distributions of amino acids chains, pH7 (kcal/mol) [54].

	Amino Acid	Feature Value		Amino Acid	Feature Value
1	I	4.92	11	Y	-0.14
2	L	4.92	12	T	-2.57
3	V	4.04	13	S	-3.40
4	P	4.04	14	H	-4.66
5	F	2.98	15	Q	-5.54
6	M	2.35	16	K	-5.55
7	W	2.33	17	N	-6.64
8	A	1.81	18	E	-6.81
9	C	1.28	19	D	-8.72
10	G	0.94	20	R	-14.92

Given:

Amino_Acid [] = {C, Q, E, G, H, I, L, K, M, F, P, S, T, W, Y, V,...} and

Hydrophobicity_Index [] = {1.28, -5.54, -6.81, 0.94, -4.66, 4.92, 4.92, -5.55, 2.35, 2.98, 4.04, -3.40, -2.57, 2.33, -0.14, 4.04,...},

$$hydrophobicity\_matrix[i][j]=\frac{abs(hydrophobicity\_index[i]-hydrophobicity\_index[j])}{20}$$

where the denominator 20 is used to convert the data range into [0, 1]. Hydrophobicity matrix [3,4] means the absolute value of the difference of the hydrophobicity indices of two amino acids E (-6.81) and G (0.94). With the range adjustment, now we obtain 0.2935.

In the case of structure/function families classification of protein sequences, various hydrophobicity scales were thoroughly examined by David [40]. He showed the effectiveness of numerous hydrophobicity scales, and concluded that the Rose scale [41] was superior to all others when used for protein structure prediction. The Rose scale is correlated to the average area of buried amino acids in globular proteins and is shown in Table 8[41]. However, Korenberg et al. [36], stated several key drawbacks with Rose scale. As it is not a one-to-one mapping, different amino acid sequences can have identical hydrophobicity profiles. Also, the scale covers a narrow range of values, while causing some amino acids to be weighted more heavily than others. To overcome these problems, the SARAH1 scale, five bits "state" representation for amino acid was introduced by Korenberg et al.

**Table 8:** Rose hydrophobicity scale [55]

	Amino Acid			Amino Acid	Feature Value
1	A	0.74	11	L	0.85
2	R	0.64	12	K	0.52
3	N	0.63	13	M	0.85
4	D	0.62	14	F	0.88
5	C	0.91	15	P	0.64
6	Q	0.62	16	S	0.66
7	E	0.62	17	T	0.70
8	G	0.72	18	W	0.85
9	H	0.78	19	Y	0.76
10	I	0.88	20	V	0.86

SARAH1 assigns each amino acid a unique five-bit signed code where exactly two bits are non-zero. SARAH1 ranks twenty possible amino acids according to the Rose hydrophobicity scale (Table 8). Each amino acid is assigned a five bit code in descending order of the binary value of the corresponding code. One of the benefits to using the five-bit code is that the complexity of the classifier can be significantly reduced and can arrange these numbers in thirty-two possible ways (2^5 ^= 32). If the representations with no or all ones, and those with 1 or 4 ones are removed, there are exactly twenty representations left. This leaves just enough representation to code for the twenty amino acids. In the case of window size five, a residue vector has 5*11 = 55 dimensions, which leads to less model complexity than the residue vector using orthogonal encoding (20*11 = 220 dimensions) [27].

The resulting scale in Table 9, where the right half is the negative mirror image of the left half, is referred to as SARAH1. The ten most hydrophobic residues are positive, and the ten least hydrophobic residues are negative. Korenberg et al [36]. indicated that while the above scales carry information about hydrophobicity, scales can similarly be constructed to embed other chemical or physical properties of the amino acids such as polarity, charge, *α *– *helical *preference, and residue volume.

**Table 9:** SARAH1 Scale.

	Amino Acid	Binary Code		Amino Acid	Binary Code
1	C	1, 1, 0, 0, 0	11	G	0, 0, 0, -1, -1
2	F	1, 0, 1, 0, 0	12	T	0, 0, -1, 0, -1
3	I	1, 0, 0, 1, 0	13	S	0, 0, -1, -1, 0
4	V	1, 0, 0, 0, 1	14	R	0, -1, 0, 0, -1
5	L	0, 1, 1, 0, 0	15	P	0, -1, 0, -1, 0
6	W	0, 1, 0, 1, 0	16	N	0, -1, -1, 0, 0
7	M	0, 1, 0, 0, 1	17	D	-1, 0, 0, 0, -1
8	H	0, 0, 1, 1, 0	18	Q	-1, 0, 0, -1, 0
9	Y	0, 0, 1, 0, 1	19	E	-1, 0, -1, 0, 0
10	A	0, 0, 0, 1, 1	20	K	-1, -1, 0, 0, 0

In order to create a new profile, we use the idea above in addition to the existing sequence profile generated by PSI-BLAST. As suggested in Figure 2, the less-discriminatory features in the sequence profile are removed by using the auto-associative network in order to prevent some possible problems that may be caused by the high complexity of the learner. Finally, CEH-Profile which contains selected evolutionary information and SARAH1 hydrophobicity scale is created.

### Learning in high dimensional space

Protein sequence data can be mathematically viewed as points in a high dimensional space. For example, a sequence of 10 amino acids represents a search space of 20^10 ^possibilities and requires a network of 200 inputs. In many applications, the curse of dimensionality is one of the major problems that arise when using non-parametric techniques [42].

Learning in the high dimensional space causes several important problems. First, the good data fitting capacity of the flexible "model-free" approach often tends to fit the training data very well and thus, have a low bias. However, the potential risk is the overfitting that causes high variance in generalisation. In general, the variance is shown to be a more important factor than the learning bias in poor prediction performance [43]. Second, with the high dimensional data, as the number of hidden nodes of the network is severely increased, it eventually leads to an exponential rise in computational complexity. A high complexity model generally shows a low bias but a high variance [44]. On the other hand, a model with low complexity shows a high bias but a low variance. Hence, a good model balances well between model bias and model variance. This problem is generally regarded as the term "*bias-variance tradeoff*".

### Semi-parametric modelling for the bias-variance tradeoff

One of the solutions to the problems above is so-called semi-parametric modelling. Semi-parametric models take assumptions that are stronger than those of non-parametric models, but are less restrictive than those of parametric model. In particular, they avoid most serious practical disadvantages of non-parametric methods at the price of an increased risk of specification errors.

The proposed model, Adaptive-LEKM takes a form of the semi-parametric model and it finds the optimal trade-off between parametric and non-parametric models. Thus, it can take advantages of both models while effectively avoiding the curse of dimensionality. The Adaptive-LEKM contains the evolutionary information represented within the local model. Its global model works as a collaborative filter that transfers the knowledge amongst the local models in formats of the hyper-parameters. Here, as a local model of Adaptive-LEKM, an effective data compression technique is used for the data localisation. The local model contains an efficient vector quantisation method.

### Vector quantisation for locality-effectiveness

Vector Quantisation (VQ) is a lossy data compression technique based on the principle of book coding. Its basic idea is to replace with key values from an original multidimensional vector space into values from a discrete subspace of lower dimension. The lower-space vector requires less storage space and the data is thus compressed.

Consider a training sequence consisting of *M *source vectors, *T *= {*x*_1_, *x*_2_,..., *x*_*m*_}. *M *is assumed to be sufficiently large and so that all the statistical properties of the source are captured by the training sequence. We assume that the source vectors are *k*-dimensional, *X*_*m *_= (*x*_*m*,1_, *x*_*m*,2_,..., *x*_*m*, *k*_), *m *= 1, 2,..., *M*. These vectors are compressed by choosing the nearest matching vectors and form a codebook consisting the set of all the codevectors. *N *is the number of codevectors, *C *= {*c*_1_, *c*_2_,..., *c*_*n*_} and each codevector is k-dimensional, *c*_*n *_= (*c*_*n*,1_, *c*_*n*,2_,..., *c*_*n*, *k*_), *n *= 1, 2,..., *N*. The representative codevector is determined to be the closest in Euclidean distance from the source vector. The Euclidean distance is defined by:

$$d(x,c_{i})=\sqrt{\sum_{j=1}^{k}{\left(x_{j}-c_{ij}\right)}^{2}}$$

where

*x*_*j *_= the *j*^*th *^component of the source vector,

*c*_*ij *_= the *j*^*th *^is components of the codevector *c*_*i*_.

*S*_*n *_is the nearest-neighbour region associated with codevector *c*_*n*_, and the partitions of the whole region are denoted by *P = {S*_1_, *S*_2_,..., *S*_*N*_}. If the source vector *X*_*m *_is in the region *S*_*n*_, its approximation can be denoted by *Q*(*X*_*m*_) = *c*_*n*_, if X_*m *_∈ S_*n*_. The *Voronoi *region is defined by:

*V*_*i *_= {*x *∈ *R*^*k*^: ||*x *- *c*_*i*_|| ≤ ||*x *- *c*_*j*_||, *for all j *≠ *i*}

**Figure 3:**
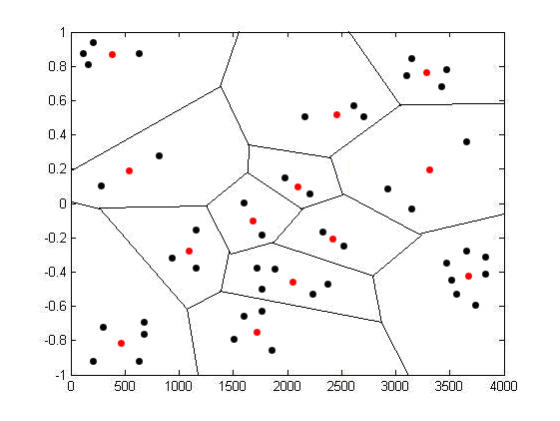
Two dimensional LBG Vector Quantisation.

As depicted in Figure 3, the training vectors falling in a particular region are approximated by a red dot associated with that region. To find the most optimal *C *and *P*, vector quantisation uses a square-error distortion measure specifying exactly how close the approximation is. The distortion measure can be given as:

$$D_{ave}=\frac{1}{Mk}\sum_{m=1}^{M}{\left\|X_{m}-Q\left(X_{m}\right)\right\|}^{2}$$

If C and P are solution to the above minimisation problem, then it must satisfy two conditions namely nearest neighbour and centroid conditions. The nearest neighbour condition indicates the sub-region *S*_*n *_should consist of all vectors that are closer to *c*_*n *_than any of the other codevectors. It is written as:

*S*_*n *_= {*x*: ||*x *- *c*_*n*_||^2 ^≤ ||*x *- *c*_*n*'_||^2 ^∀ *n*' = 1, 2,..., *N*}

The centroid condition requires the codevector *c*_*n *_should be average of all those training vectors that are in its *Voronoi Region S*_*n*_.

$$c_{n}=\frac{\sum_{Xm\in Sn}X_{m}}{\sum_{Xm\in Sn}1},n=1,2,...,N$$

### Kernel machine as an collaborating filter

As a key collaborator of Adaptive LEKM, we use an effective kernel classifier to construct the global model. SVM can be seen as a set of related supervised learning and generalised linear classifiers. The key features of SVMs are the use of kernels, the absence of local minima, the sparseness of the solution and the capacity control obtained by optimising the margin [45]. A significant advantage of SVMs is that whilst ANNs can suffer from multiple local minima, the solution to an SVM is global and unique [45,46]. SVMs are known as maximum margin classifiers since they classify their objects by minimising the empirical generalisation error and maximising the geometric margin simultaneously. Where the two classes are not separable, they map the input space into a high-dimensional feature space (where the classes are linearly separable) by using a non-linear kernel function. The kernel function calculates the scalar product of the images of two examples in the feature space. Given a *d*-dimensional input vector, *x*_*i *_= (*x*_1_, *x*_2_,..., *x*_*n*_) with two labels, *y*_*i *_∈ {+1, -1} (*i *= 1, 2,..., *N*), the hyperplane decision function of binary SVM with kernel method is written as:

$$f(x)=\mathrm{sgn}\left(\sum_{i=1}^{\ell }y_{i}a_{i}\langle \Phi (x),\Phi (x_{i})\rangle +b\right)=\mathrm{sgn}\left(\sum_{i=1}^{\ell }y_{i}a_{i}k(x,x_{i})+b\right)$$

and the following quadratic program:

maximise $W(a)=\sum_{i=1}^{\ell }a_{i}-\frac{1}{2}\sum_{i,j=1}^{\ell }a_{i}a_{j}y_{i}y_{j}k(x_{i},x_{j})$

subject to *a*_*i *_≤ 0, *i *= 1,..., ℓ, and $\sum_{i=1}^{\ell }a_{i}y_{i}=0$.

where ℓ is the number of training patters; *a*_*i *_are the parameters of the SVM; *k*(·,·) is a suitable kernel function, and *b *is the bias term.

The SVM used in the Adaptive-LEKM is the modified version of SVM^*light *^package. It uses an RBF kernel for the classification and the hyperparameters used in the SVM were optimised using a 7-fold cross-validation (Refer to Section *Overall Architecture of SiteSeek*). In order to find optimal values for the hyperparameters, a number of values were considered and tested against the newly built PS-Benchmark_1 dataset. The optimal values were chosen for the PS-Benchamrk_1 dataset were *C*: 1.5, *γ*: 0.04, and *ε*: 0.1.

### Locality-Effective Kernel Machine

In the literature, it is claimed that one of the most serious problems with SVMs is the high algorithmic complexity and extensive memory requirements of the required quadratic programming in large-scale tasks [47]. As observed in the above equation, SVM extracts worst-case examples *x*_*i *_and use statistical analysis to build large margin classifiers. However, in Adaptive-LEKM, we use the centroid vector of each *voronoi *region which can be expressed as:

$$Q_{i}(X_{m})=c_{i}=\frac{\sum_{Xm\in Si}x_{m}}{N},i=1,2,...,N$$

To construct a semi-parametric model, we substitute *Q*_*i *_(*X*) for each training sample *x*_*i *_used in the SVM decision function. The Adaptive-LEKM's approximation can be written as:

$$f(x)=\mathrm{sgn}\left(w\cdot \varphi (x)+b\right)=\mathrm{sgn}\left(\sum_{i=1}^{\ell }y_{i}a_{i}k(x,c_{i})+b\right)$$

and the following quadratic program:

maximise $W(a)=\sum_{i=1}^{\ell }a_{i}-\frac{1}{2}\sum_{i,j=1}^{\ell }a_{i}a_{j}y_{i}y_{j}k(Q_{i}(x),Q_{j}(x))$

subject to *a*_*i *_≥ 0, *i *= 1,..., ℓ, and $\sum_{i=1}^{\ell }a_{i}y_{i}=0$.

The SVM is considered as a purely non-parametric model, whereas the Adaptive-LEKM can be considered as semi-parametric model as it adopts the method of grouping of the associated input vectors in each class *i*. Hence, the performance of proposed model has some advantages in comparison to the pure parametric models and pure non-parametric models in terms of learning bias and generalisation variance especially on high dimensional protein datasets.

As the Adaptive-LEKM uses the centroid vector of each nearest neighbour region, we can obtain the optimal representations by finding right size of each sub regions. In other words, a good trade-off between parametric and non-parametric can be found by adjusting the size of each sub-region. If the feature space is partitioned to too many sub-regions, the model becomes closer to non-parametric model. So, it is eventually susceptible to overfitting which causes high model variance problem. Contrarily, if the space is divided into too small number of regions, the codevectors cannot correctly represent the original dataset (as they miss too much information). And the model eventually produces high leaning bias. Hence, it is crucial to find a good trade-off between the parametric and non-parametric models.

In Adaptive-LEKM, the size of *Voronoi regions *is continually changed until they find the most optimal trade-off between model bias and generalisation variance. Eventually, the global model is able to transfer the knowledge amongst the optimised local models in formats of the hyper-parameters. Our method which includes selection of the corresponding output *y*_*i *_and the finding optimal size of the associated input regions in each class *i *is shown to be effective in finding an optimal trade-off between model bias and variance. However, if we assume that a centroid vector is calculated by its associated cluster and the cluster contains noise vector, the centroid vector may incorrectly represent its associated cluster or regions. Hence, in our method, an auto-associative neural-network [48] is used to perform non-linear mapping from x to x' to eliminate noise or less-discriminatory features that can impede recognition.

### Auto-associative network for nonlinear dimensionality reduction

**Figure 4:**
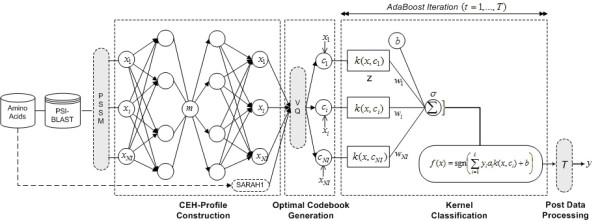
SiteSeek Basic Architecture.

For the non-linear mapping, we use Scholz's [49] standard auto-associative network as also widely known as non-linear principal component analysis (NLPCA). The auto-associative network contains three hidden layers between the input and output layers namely encoding, bottleneck and decoding units. In the hidden units as depicted in Figure 4, the input signals are transferred to the "encoding" neurons in the first hidden units. The hyperbolic tangent function is used as the transfer function here, and again when the signal moves from the "bottleneck" neuron in the second hidden layer to the "decoding" neurons in the third hidden layer. Data compression is achieved by the bottleneck, with the single bottleneck neuron giving the leading non-linear principal component. The numbers of encoding and decoding neurons are adjustable for the optimal fit, however, are set the same for simplicity. The auto-associative network in Figure 4 with 3, 4, 1, 4 and 3 neurons in its 5 layers will be referred to as a 3-4-1-4-3 model.

### AdaBoost for the network tuning

In order to maximise the performance of the Adaptive-LEKM, we utilised a network boosting method called Adaptive Boosting (AdaBoost). In general, boosting is known as a technique to improve the performance of any base machine learning algorithms. The AdaBoost algorithm was proposed by Freund and Schapire [50] and it was shown to be a solution for many practical difficulties of previous boosting algorithms. Boosting combines weak learners to find a highly accurate classifier or better fit for the training set [51]. In this study, the AdaBoost was modified for the LEKM for the network boosting. As observed in our experiments, the modified AdaBoost was tested with the LEKM and showed that it can fit into its architecture for more accurate prediction of phosphorylation sites. A standard boosting algorithm can be written as:

Given: (*x*_1_, *y*_1_),...,(*x*_*NV*_, *y*_*NV*_) where *x*_*i *_∈ *X*, *y*_*i *_∈ *Y *= {-1, +1}

Initialise *D*_1_(*i*) = 1/*NV*

For *t *= 1,...., *T*:

- Find the classifier *h*_*t *_: *X *→ {-1, +1} that minimises the error with respect to the distribution *D*_*t*_

$h_{t}=\arg\underset{h_{j}\in H}{\min}\varepsilon_{j}$ where $\varepsilon_{j}=\sum_{i=1}^{NV}D_{t}(i)[y_{i}\neq h_{j}(x_{i})]$

- Get weak hypothesis *h*_*t *_: *X *→ [0, 1]

- Choose *a*_*t *_∈ *R*, typically $a_{t}=\frac{1}{2}in\frac{1-\varepsilon_{t}}{\varepsilon_{t}}$ where *ε*_*t *_is the weighted error rate of classifier *h*_*t*_

- Update:

$$D_{t+1}(i)=\frac{D_{t}(i)\exp(-a_{t}y_{i}h_{t}(x_{i}))}{Z_{t}}$$

where

*NV *= total number of training vectors,

*X *= a domain or instance space of each *x*_*i *_belong to,

*Y *= a label set of each label *y*_*i*_,

*Z*_*t *_= a normalisation factor (chosen so that *D*_*t*+1_will be a distribution),

*R *= its sign is the predicted label {-1, +1}.

Output the final hypothesis:

$$H(x)=sign\left(\sum_{t=1}^{T}a_{t}h_{t}(x)\right)$$

In the training set, each *x*_*i *_belongs to a domain X, and each label *y*_*i *_is in a label set Y. Here, the Y should be {-1, +1} as phosphorylation sites are indicated as positive (+1) or negative (-1) values only. After selecting an optimal classifier *h*_*t *_for the distribution *D*_*t*_, the examples *x*_*i *_that the classifier *h*_*t *_identified correctly are weighted less and those that it identified incorrectly are weighted more. Therefore, when the algorithm is testing the classifiers on the distribution *D*_*t*_+1, it will select a classifier that better identifies those examples that the previous classifier missed. At each iteration, the AdaBoost embedded in Adaptive-LEKM constructs weak learners based on this method called *weighted examples*.

### Overall Architecture of SiteSeek

As illustrated in Figure 4, SiteSeek contains four main components. First, given amino acids sequences, SiteSeek utilise PSI-BLAST to generate Position Specific Scoring Matrix (PSSM) with an e-value threshold for inclusion of 0.001 and six search iterations of non-redundant (*nr*) sequence database. The PSSM has 20 × *N *elements, where *N *is the length of the target sequence and each element represents the log-likelihood of a particular residue substitution based on a weighted average of BLOSUM62 [52] matrix scores for a given alignment position in the template.

Second, compact PSSM is generated by using the standard auto-associative network. As all the possible less discriminatory features and noises are expected to be eliminated in this step, it is crucial in generating the optimal codebook in the following step. To construct CEH-Profile, SARAH1 scales are computed from the amino acid chains in PS-Benchmark_1 dataset and are added to the compact PSSM. The CEH-Profile which contain compact PSSM and SARAH1 scales was all normalised to fall in the interval [-1, 1] by using following algorithm.

pn = 2*(p-minp)/(maxp-minp) - 1

where

p = R × Q matrix of input vectors,

minp = R × 1 vector containing minimums for each p,

maxp = R × 1 vector containing maximums for each p.

Third, to find the most optimal parameter *C *and *P *as for the solution of the minimisation problem, vector quantisation uses the given distortion measure (See Section *Vector Quantisation for Locality-Effectiveness*). As discussed in Section *Locality-Effective Kernel Machine*, the modified vector quantiser in Adaptive-LEKM finds the most suitable number of *Voronoi regions *by considering model bias and generalisation variance generated in each iteration so that the codebook is generated for the classification.

Fourth, our kernel machine uses the resulting codebook and performs its classification tasks. For the fair comparison of our proposed model, we adopted a seven fold cross-validation scheme for the model evaluation. The PS-Benchmark_1 dataset was divided into seven sub-samples. One of the sub-samples was used for testing and the rest six were used for training. The testing was conducted seven times for each model. When multiple random training and testing experiments were performed, a model was formed from the six sub-samples (training samples). The estimated prediction accuracy is the average of the prediction accuracy over seven different datasets for the models. We used the window size of 9 for tyrosine and threonine, and 11 for serine sites [15]. A window size of 9 means 19 amino acids with the tyrosine, threonine or serine site is located at the centre of the window.

Finally, with the threshold *T*, the final predictions are simulated from the raw output generated by Adaptive-LEKM. During the post-processing of the network output, as the network generates the raw outputs which have many local peaks, we modified Liu and Rost's [53] method to filter these raw outputs. First, we determined the threshold for each network outputs according to the length (*L*) of the protein and to the distribution of raw output values for all residues in that protein. We compiled the 96th percentile of the raw output *T*_1 _and set the threshold *T *to

$$T=[\begin{array}{ll} \max(T_{1},60) & \text{for L}\leq 100\\ \max(T_{1},30) & \text{for L}\leq 200\\ T_{1} & \text{for L}>200 \end{array}$$

*T *was set to the threshold that divides phosphorylation site and others. If the value of a residue is above the threshold, the residue is regarded as phosphorylation site. Second, we assigned the central residue as a phosphorylation site if three or more residues were predicted as a phosphorylation site. And all parameters for these filters were developed using the validation set only.

The performance of SiteSeek is measured by the accuracy (Ac: the proportion of true-positive and true-negative residues with respect to the total positives and negatives residues), the sensitivity (Sn: the proportion of correctly predicted phosphorylation site residues with respect to the total positively identified residues), the specificity (Sp: the proportion of incorrectly predicted site residues with respect to the total number of phosphorylation site residues) and correlation coefficient (Cc: It balances positive predictions equally with negative predictions and varies between -1 and 1.). Cc reflects a situation in that a method which predicts every residue to be positive, shows prediction accuracy of 100% in detecting positive sites, however 0% accuracy for negative residues. Hence, high value of Cc means that the model is regarded as a more robust prediction system. In addition to the four measures above, the performance of each model is additionally measured by Type I and Type II Error rates as incorrectly predicted residues can be as valuable as the correctly predicted residues for further modification of the model. Type I Error means experimentally verified unmodified sites that are predicted (incorrectly) to be modified; And Type II Error indicates experimentally verified modified sites that are predicted (incorrectly) to be unmodified. The Sn, Sp, Ac and CC can be expressed in terms of true positive (TP), false negative (FN), true negative (TN) and false positive (FP) predictions.

$$\begin{array}{ccc} Sn=\frac{TP}{TP+FN^{'}}, & Sp=\frac{TN}{TN+FP^{'}}, & Ac=\frac{TP+TN}{TP+FP+TN+FN'} \end{array}$$

and

$$Cc=\frac{\left(TP\times TN)-(FN\times FP\right)}{\sqrt{\left(TP+FN)\times (TN+FP)\times (TP+FP)\times (TN+FN\right)}}$$

The stepwise procedure we have performed can be summarised as follows:

(1) Data collection, building a new dataset and pre-processing datasets.

(2) Profiles construction such as PSSM, Orthogonal encoding, and Sarah1.

(3) Hold-out method was performed to divide the combined dataset into 7 subsets (training and testing sets).

(4) CEP-Profile construction for each subset using the auto-associative network.

i. The auto-associative network was built with five layers in 3-4-1-4-3 model.

ii. Activation Function Selection: Hyperbolic tangent function.

iii. The original data dimension is reduced into dm_17 (compact_PSSM).

iv. Sarah1 scale is added to the compact_PSSM.

(5) The information obtained in (2) and (3) were combined and normalised to fall in the interval [-1, 1] to be fed into networks.

(6) Assign target levels in each profile.

i. Positive (1) for phosphorylation site residues and negative (-1) for non-site residues. (For tyrosine or threonine sites, nine neighbouring residues are assigned positive (1) whereas eleven neighbouring resides are positive for serine sites).

(7) Train each model on training set to create a model

(8) Simulate each model on test set to obtain predicted outputs.

(9) Post-processing was performed to find predicted phosphorylation sites locations.

The procedure from (7) to (9) is performed iteratively until we obtain the most suitable kernel and the optimal hyperparameters for Adaptive-LEKM for the given benchmark dataset.

## Abbreviations

Adaptive-LEKM: Adaptive Locality-Effective Kernel Machine, AGC: cAMP-dependent protein kinase/protein kinase G/protein kinase C extended family, ANN: Artificial Neural Network, CAMK: Calcium/calmodulin-dependent Kinase, CEH-Profile: Compact Evolutionary and Hydrophobicity Profile, CDK: Cyclin-dependent Kinase, CK2: Casein Kinase II, CMGC: Cyclin-dependent kinase-like Kinase, DT: Decision Tree, GRNN: General Regression Neural Network, KLR: Kernel Logistic Regression, kNN: Kernel Nearest Neighbour, MLP: Multi-Layered Perceptron, OE: Orthogonal Encoding, PK: Protein Kinase, PKA: cAMP-dependent protein Kinase, PKC: Protein Kinase C, PSI-BLAST: Position Specific Iterated Basic Local Alignment Search Tool, PSSM: Position Specific Scoring Matrix, RBFN: Radial Basis Function Neural Network, SARAH1: Simultaneously Axially and Radially Aligned Hydrophobicities, SVM: Support Vector Machine, TK: Tyrosine Kinase.

## Conflict of interests

The authors declare that they have no competing interests.

## Authors' contributions

PDY developed and implemented the new model (Adaptive-LEKM) and profile (CEH-Profile), and drafted the manuscript. YSH prepared datasets, programming in C++, Shell and Perl scripts, and interpreted the results with PDY. BBZ and AYZ edited the manuscript and introduced the problem initially.
